# The Lords' Committee

**Published:** 1891-02-28

**Authors:** 


### THE LORDS' COMMITTEE.

Charing Cross Hospital came out well before the Lords' Committee last week, and the witnesses all gave evidence in an excellent manner. Mr. A. E. Reade (the Secretary of the Charing Cross Hospital) was called, and, in reply to the chairman, stated that he had been nine years secretary. The hospital, which was free, was founded in 18.0, in Villiers Street, whence it was removed in 1831 to its present situation. It was opened in 1834. Although Governors' letters were issued, patients having them had no preference over others. The test of admission was whether the patient was a poor person or was a suitable case for hospital relief. With the exception of a few beds kept vacant for accidents, all the available working beds, numbering 175, were nearly always in use. He should think that the medical relief in that part of the metropolis was sufficient for the requirements. There were a few dispensaries in the neighbourhood. If there were any complaints from the patients the fact would be made known to him, and he should sift the matter to the bottom. Although he had been there nine years he could not remember having heard of a complaint. The nursing staff comprised ten Sisters, seventeen nurses, and twenty-four probationers. Since March, 1889, the hospital authorities had undertaken the nursing themselves. Previously it was done by the St. John's House Sisterhood. The dual control was, however, found to be unsatisfactory. The Sisters were on duty eight and a-half hours daily, and on one Sunday five and the next Sunday nine hours. The nurses and probationers were on duty ten hours daily, and on one Sunday nine, and the next Sunday six hours. There was no pension provided for them, but the council had that question under their consideration. The nurses received £22, increasing to £25 a-year, with uniform, and 2s. 6d. a-week for washing. The probationers had no salary the first year, £15 the second, and £20 the third year, with uniform and washing. Lord Cathcart, after putting a few questions to the witness, said: "A noble Lord here present and myself paid you a surprise visit last evening, and went over the whole of your establishment, and you will not be sorry to hear that in the opinion, I believe, of the noble Lord, certainly in my own opinion, everything we saw, considering the limited nature of your site, was most satisfactory." Mr. Stanley Boyd, Dean of the Medical School, said the students numbered 228, and the school was steadily increasing. The total gross revenue was £4,070. Mr. Boyd expressed himself in favour of the present system of medical education, save in the absence of a uniform standard for the preliminary examination. He also said he saw no reason why this preliminary examination should not be in the hands of the State, and include botany, zoology, and chemistry. Mr. Boyd took occasion to demur at some slighting remarks made by Sir Andrew Clark about the teaching powers in physiology and chemistry of the Charing Cross School. Dr. Frederick Willcocks agreed with Mr. Boyd as to the present system of medical education. As a physician to out-patients for nine years, he did not consider that department of the hospital abused, and he considered valuable experience was gained there by the students. He found that "gentlemen of colour" made excellent medical students.

On Monday, Mr. Ryan, Secretary of St. Mary's Hospital, was examined, and stated that hospital accommodation in Paddington was deficient. Mr. Ryan's most important remarks referred to the figures contained in the Charity Organisation Society's memorandum as to the hospital expenses and the comparative cost per bed. That was a most fallacious comparison, extremely misleading, and productive of considerable mischief. A hospital taking a large number of cancer cases increased its expenses; and if the proportion of children in a general hospital was large, the expense per head was less. The extent of the maternity department would also considerably affect the out-patient department expenses. Some hospitals were rated at their full value, while others would only pay a small fraction. The nursing staff consisted of the Matron, one Night Superintendent, ten Sisters, twenty-six staff nurses, and twenty-three probationers. The proportion of beds to the nurses on day duty was about seven. The Sisters were off duty twice a week, from five to half-past ten, once a week from seven to half-past ten, every other Sunday from three to half-past ten, and on Saturday from two till half-past ten. Their holidays were one day per month and one calendar month per annum. The staff nurses' hours were seven to nine p.m. They wee off duty weekly from five, and once weekly from seven, and every other Sunday from three p.m. Their holidays were one day per month, and one calendar month per annum. The probationers' hours were from seven to half-past eight, off duty two hours daily, from ten to twelve in one week, and from half-past two to half-past four in the other week. Their holidays were once a month, and annually three weeks. From his experience of the Metropolitan Asylums Board he was against any central authority for hospitals. Mr. H. W. Page, a surgeon of the hospital, stated that the students numbered 300. Owing to the competition of other schools there had last year been a diminution in the number of students. The average income of the school was £4,500. The largest amount ever received was £5,900 in 1887-8. The cost of keeping the school going was about £3,400, and the salaries paid were £1,100. In reply to the question whether he wished any reform in medical education, the witness replied that it might be improved in various ways. For instance, before the student went in for his medical education it would be beneficial if he had already been instructed in botany, chemistry, and physics. If the individuality of the schools were not lost, it might be well to have some sort of central educational institution, for there was a great waste of money and energy in maintaining the fully equipped medical schools in London, while it might be beneficial to have some central authority—perhaps connected with the County Council—having a voice in deciding whether or not hospitals should be erected in particular places. They should, however, be kept quite free from Government interference. Mr. Malcolm Morris, physician of the skin department of the same hospital, gave evidence in favour of utilising the poor-law infirmaries for medical teaching, as being beneficial to both patients and students. No hospital should be formed without a licence. He advocated uniformity in hospital management as well as in their accounts.

